# Association between Integration Policies and Immigrants’ Mortality: An Explorative Study across Three European Countries

**DOI:** 10.1371/journal.pone.0129916

**Published:** 2015-06-12

**Authors:** Umar Z. Ikram, Davide Malmusi, Knud Juel, Grégoire Rey, Anton E. Kunst

**Affiliations:** 1 Department of Public Health, Academic Medical Center, University of Amsterdam, Amsterdam, the Netherlands; 2 Agència de Salut Pública de Barcelona (IIB-Sant Pau), Barcelona, Spain; 3 CIBER Epidemiología y Salud Pública, Barcelona, Spain; 4 National Institute of Public Health, University of Southern Denmark, Copenhagen, Denmark; 5 INSERM, CépiDc, Le Kremlin-Bicxtre, France; National Institute of Health, ITALY

## Abstract

**Background:**

To integrate immigrants into their societies, European countries have adopted different types of policies, which may influence health through both material and psychosocial determinants. Recent studies have suggested poorer health outcomes for immigrants living in countries with poorly rated integration policies.

**Objective:**

To analyse mortality differences of immigrants from the same country of origin living in countries with distinct integration policy contexts.

**Methods:**

From the mortality dataset collected in the Migrant Ethnic Health Observatory (MEHO) project, we chose the Netherlands (linked data from 1996-2006), France (unlinked; 2005-2007) and Denmark (linked; 1992-2001) as representatives of the inclusive, assimilationist and exclusionist policy models, respectively, based on the Migrant Integration Policy Index. We calculated for each country sex- and age-standardized mortality rates for Turkish-, Moroccan- and local-born populations aged 20-69 years. Poisson regression was used to estimate the mortality rate ratios (MRRs) for cross-country and within-country comparisons. The analyses were further stratified by age group and cause of death.

**Results:**

Compared with their peers in the Netherlands, Turkish-born immigrants had higher all-cause mortality in Denmark (MRR men 1.92; 95% CI 1.74-2.13 and women 2.11; 1.80-2.47) but lower in France (men 0.64; 0.59-0.69 and women 0.58; 0.51-0.67). A similar pattern emerged for Moroccan-born immigrants. The relative differences between immigrants and the local-born population were also largest in Denmark and lowest in France (e.g., Turkish-born men MRR 1.52; 95% CI 1.38-1.67 and 0.62; 0.58-0.66, respectively). These patterns were consistent across all age groups, and more marked for cardiovascular diseases.

**Conclusions:**

Although confounders and data comparability issues (e.g., French cross-sectional data) may affect the findings, this study suggests that different macro-level policy contexts may influence immigrants’ mortality. Comparable mortality registration systems across Europe along with detailed socio-demographic information on immigrants may help to better assess this association.

## Introduction

### Immigrants’ integration policy models in Europe

In the context of decolonisation and economic expansion in the decades following World War II, Western European countries welcomed large numbers of immigrants to meet the increasing labour demand. Governments adopted legislations and policies with the aim to control the influx and successfully integrate these immigrants and their families into the new host environment. Interestingly, no shared set of policies was implemented, rather the policies tended to differ by country, probably due to different political ideologies, national histories and cultural traditions.

Several authors have identified, with different names, three models of integration policies in Europe. First, the “ethnic minorities”, “multicultural” or “individualistic-civic” model combines social and political tolerance and respect of cultural differences with facilities to acquire citizenship through residence or place of birth (*ius soli*), with the UK, Netherlands and Sweden consistently classified in this group. Second, the “guest worker”, “differential exclusionist” or “collectivistic-ethnic” model, with Germany as historical prototype, assumes a conjunctural presence of immigrants based on the labour market needs. This model bases citizenship on ancestry (*ius sanguinis*), puts in place few active integration policies, and goes along with low levels of social and political tolerance. Third, the “assimilation” or “collectivistic-civic” model, with France as an example, facilitates citizenship through the *ius soli* principle, but is not keen on public manifestations of cultural differences and requires adhesion to republican values [1,2].

It is important to note that through confrontation to similar problems, policy orientations have sometimes changed and increasingly converged, especially in the European Union context. For example, Germany has been very similar in practice to France, and has reformed and opened its nationality law [3,4]. It might therefore be important to characterise policies as they have developed in practice. An example of country typology based on a systematic evaluation of current policies is that proposed by Meuleman [5] using the Migrant Integration Policy Index (MIPEX), an up-to-date comparison across Europe of policies related to immigrant populations based on the assessment by independent scholars and practitioners in migration law of the country’s publicly available documents [6]. Through a latent class analysis of the scores on specific dimensions of MIPEX 2007 edition, Meuleman identified three groups: a more inclusive one scoring highest on all dimensions and including the three traditional representatives of the multicultural model, among others; one with low scores, consisting of Austria, Denmark, Greece and the Eastern bloc, that shares characteristics of the differential exclusionist model; and a small cluster that the author considers an evolution of the assimilationist model, with scores similar to exclusionist countries on residence and access to labour market but similar to inclusive countries on nationality and political participation (including France and “former exclusionists” like Germany and Switzerland).

### From integration policy to immigrants’ health and mortality

As policies largely determine the social environments in which individuals work and live, these contrasting integration policies may have created disparate socioeconomic contexts for immigrants, including employment opportunities, income and housing conditions. These factors are known to have an impact on morbidity and mortality (i.e., the material pathway) [7,8] and to contribute to ethnic inequalities in mortality [9,10]. In addition, the institutional arrangements and policies may be reciprocally linked to the host population’s attitudes towards immigrants [2,5,11], all of which might affect immigrants’ health through chronic negative daily stressors such as experiences of social exclusion, intolerance and discrimination (i.e., psychosocial pathway) [7,12]. In the US, studies have indicated an impact of racial discrimination policies and their abolition on mortality of racial minorities [13,14]. Given these potential pathways, immigrants’ health might differ according to the country of residence integration policies. In two recent European studies, the global MIPEX score failed to show a relationship with depression in immigrants [12], while it was found to be related with a smaller disadvantage as compared to non-migrants in subjective wellbeing [15]. In a more recent study, the policy model approach, as proposed by Meuleman with the three types, gave more consistent results. It found that in countries with exclusionist policy model immigrants had poorer self-rated health and larger inequalities, as compared to countries with other policy models. [16].

In the present study we aimed to explore whether such relationship could be observed with all-cause mortality and various causes of death. Based on the available cross-country mortality data from the Migrant and Ethnic Health Observatory (MEHO) project [17], we selected countries belonging to the different typologies of integration policies according to the MIPEX 2007 analysis [5]: Netherlands as “inclusive”, France as “assimilationist”, and Denmark as “exclusionist”. For these three countries we had a substantial representation of immigrants from two countries of origin—Turkey and Morocco (see Table 1 for person-years at risk in each country). Across the three countries, these immigrants had similar background (e.g., little education, unskilled, rural origin), and similar reasons for migration, being recruited to fill up the Western European labour shortages in 1960–1970s with subsequent family reunifications [18,19]. By restricting the comparison to immigrants from the same country of origin, we reduced the differences potentially attributable to pre-migration exposures, with a relevant influence on immigrants’ mortality [17,20].

**Table 1:** Person-years at risk (PYR), total deaths, and age-standardized mortality rate (ASMR) in local-born, Turkish-born and Moroccan-born immigrants aged 20–69 years in three European countries, stratified by sex.

	Men			Women		
	PYR	Total deaths (n)	ASMR (per 100,000 PY)	PYR	Total deaths (n)	ASMR (per 100,000 PY)
*Local-born*						
Netherlands	48,030,138	206,576	364.0	47,104,706	129,458	224.5
France	48,978,090	235,814	417.0	50,729,854	108,660	177.2
Denmark	17,369,353	100,760	513.6	17,103,404	67,070	325.0
*Turkish-born*						
Netherlands	1,113,842	3,319	424.6	997,062	1,254	203.3
France	340,123	743	271.8	287,037	242	121.3
Denmark	105,719	431	897.0	88,042	173	467.3
*Moroccan-born*						
Netherlands	926,149	2,183	285.8	771,189	882	183.8
France	1,130,385	3,967	261.7	1,053,177	1,733	141.2
Denmark	21,442	73	811.5	14,794	17	232.2

PYR = person-years at risk. PY = person-years. ASMR = age-standardized mortality rate.

We hypothesized that all-cause mortality levels and the mortality gap with the local-born would be the highest for immigrants residing in Denmark, followed by France and then Netherlands. To formulate our hypotheses on how this relationship would vary by main cause of death, age and sex, we drew parallels with the literature on socioeconomic inequalities in mortality, suggesting that socioeconomic inequalities tend to be the largest for injury-related causes (e.g., homicide and suicide), cardiovascular diseases (CVD), and respiratory diseases [21]. In addition, socioeconomic inequalities in mortality are generally consistent across age and sex, but largest among men younger than 45 years [22]. Thus, we hypothesized the former association between country of residence and immigrants’ mortality to be stronger in younger men and for the abovementioned causes.

## Methods

### Study design, population and data sources

Population and mortality data were used from the MEHO project. Detailed information on data acquisition has been published elsewhere [23]. We drew data from the Netherlands, France, and Denmark. We included the local-born populations and two immigrant populations—i.e., Turkish- and Moroccan-born—residing in the three countries and aged 20–69 years, since relatively few deaths were observed among immigrants aged below and above this range.

In the Netherlands and Denmark, linked data were collected using linkages between records of the population register and subsequent mortality data. An open cohort design was used, so participant could enter and exit the study at any point in time during the follow-up period. Data from Denmark were collected between 1992–2001 and from the Netherlands between 1996–2006. In France, unlinked data were used; we derived numbers of deaths by country of birth, sex, and age from the national mortality register and calculated the corresponding person-years at risk (PYR) using population census information. Data from France were collected between 2005–2007.

### Ethics statement

Since we used anonymized data, no ethical approval was required.

## Variables

All-cause mortality and main causes of death were assessed. We included the following main causes of death (with International Classification of Diseases codes in brackets): suicide (ICD-9 E950-959; ICD-10 X60-X84, Y87.0); homicide (ICD-9 E960-E969; ICD-10 X85-Y09, Y87.1); CVD (ICD-9 390–459, 250; ICD-10 I0-I99, E10-E14); respiratory diseases (ICD-9 161–163, 165, 487, 480–486, 490–494, 496; ICD-10 J40-47, J10-18, C30-34, C39); infectious diseases (ICD-9 279.5, 001–139; ICD-10 B20-B24, A00-B99); cancer (ICD-9 140–239 [excluding 161–163, 165]; ICD-10 C00-D48 [excluding C30-34, C93]); unintentional injuries (ICD-9 E800-E915; ICD-10 V01-V99, W00-X59); and other causes (rest).

Country of residence, country of birth, sex and age (categorised into five-year age groups) were the other variables used.

### Data analysis

We calculated the age-standardised mortality rates (ASMR) by sex, country of residence and country of birth applying direct standardisation using the WHO World Standard Population.

Poisson regression was used to estimate the age-adjusted mortality rate ratios (MRRs). The MRRs were calculated in two ways. First, the mortality rates were compared within a population residing across the three countries (cross-country comparison between peers), with those residing in the Netherlands, the country with the best integration policy score, as the reference group. Second, we compared the mortality rates of Turkish- and Moroccan-born immigrants with those of the local-born populations within each country (within-country comparison). The models used the number of deaths as the dependent variable; five-year age groups and country of residence/birth (depending on the comparison) as independent variables; and PYR as the offset variable. We first used sex-stratified models. Second, similar models were employed but with further stratification for the age groups 20–44 and 45–69 years. Finally, we ran separate models for the main causes of death, adjusted for age and sex—no important differences between men and women were observed (sex-specific models are presented as S1A and S1B Table). SPSS version 21.0 and Microsoft Excel 2011 were used for analysis.

## Results

In Table 1 total deaths and ASMRs are presented by sex, country of residence, and country of birth. Turkish- and Moroccan-born immigrants residing in Denmark had the highest ASMR, followed by those residing in the Netherlands and then France. This pattern was also observed in local-born women, while Dutch men had the lowest mortality rate.

Table 2 presents MRRs for all-cause mortality with cross-country and within-country comparisons. Compared with their peers in the Netherlands, Turkish- and Moroccan-born had higher mortality in Denmark—the MRRs were 1.92 (95% confidence interval [CI] 1.74–2.13), 2.11 (1.80–2.47), 2.13 (1.68–2.69), and 1.39 (0.86–2.25), respectively. By contrast, mortality among Turkish- and Moroccan-born immigrants residing in France was consistently lower.

**Table 2:** All-cause mortality rate ratios (MRRs) in Turkish- and Moroccan-born immigrants in three European countries, compared with the local-born population^a^ and with peers in the Netherlands, stratified by sex.

	Men				Women			
	vs. peers		vs. local-born		vs. peers		vs. local-born	
	MRR^b^	95% CI	MRR^b^	95% CI	MRR^b^	95% CI	MRR^b^	95% CI
*Local-born*								
Netherlands	1.0	ref.			1.0	ref.		
France	1.13	1.12–1.14			0.78	0.77–0.78		
Denmark	1.40	1.39–1.41			1.46	1.45–1.48		
*Turkish-born*								
Netherlands	1.0	ref.	1.17	1.13–1.21	1.0	ref.	0.89	0.84–0.94
France	0.64	0.59–0.69	0.62	0.58–0.66	0.58	0.51–0.67	0.62	0.54–0.70
Denmark	1.92	1.74–2.13	1.52	1.38–1.67	2.11	1.80–2.47	1.34	1.15–1.55
*Moroccan-born*								
Netherlands	1.0	ref.	0.81	0.78–0.85	1.0	ref.	0.83	0.77–0.88
France	0.91	0.87–0.96	0.62	0.60–0.64	0.78	0.72–0.85	0.78	0.74–0.82
Denmark	2.13	1.68–2.69	1.31	1.04–1.65	1.39	0.86–2.25	0.88	0.54–1.41

MRR = mortality rate ratios. CI = confidence interval.

^a^The reference group was the local-born population in the respective country of residence.

^b^Mortality rate ratios were adjusted for age.

Within-country comparisons showed that immigrants in Denmark had an unfavourable mortality pattern, compared to the local-born population (Table 2). For immigrants residing in the Netherlands and France, the mortality pattern tended to be more favourable. Specifically, the MRRs for Turkish-born men and women residing in Denmark were 1.52 (95% CI 1.38–1.67) and 1.34 (1.15–1.55) and for Moroccan-born men 1.31 (1.04–1.65). By contrast, in France the MRRs for both Turkish- and Moroccan men and women varied between 0.62 and 0.78. In the Netherlands only Turkish-born men had higher mortality than the local-born population (MRR 1.17; 95% CI 1.13–1.21), while others had lower mortality (MRRs varying between 0.81 and 0.89).

Table 3 presents age-stratified analysis. In both age groups mortality for immigrants was generally lower in France but higher in Denmark than their peers in the Netherlands. For Turkish-born in Denmark, the MRR was higher in the age group 45–69 years than 20–44 years, compared both with the their peers in the Netherlands and with the local-born. Cross-country comparisons, for example, showed that Turkish-born men and women in Denmark had MRRs of 2.22 (95% CI 1.98–2.49) and 2.41 (2.00–2.91), respectively, in the age group 45–69 years versus 1.28 (1.03–1.59) and 1.54 (1.13–2.10) in the younger age group. This pattern was less consistent in the Moroccan-born.

**Table 3:** All-cause mortality rate ratios (MRRs) with 95% confidence intervals in Turkish- and Moroccan-born immigrants in three European countries, compared with the local-born population^a^ and with peers in the Netherlands, stratified by sex and age group.

	Men				Women			
	20–44 years		45–69 years		20–44 years		45–69 years	
	MRR^b^ vs. peers	MRR^b^ vs. local-born	MRR^b^ vs. peers	MRR^b^ vs. local-born	MRR^b^ vs. peers	MRR^b^ vs. local-born	MRR^b^ vs. peers	MRR^b^ vs. local-born
*Local-born*								
Netherlands	1.0 (ref.)		1.0 (ref.)		1.0 (ref.)		1.0 (ref.)	
France	1.49 (1.47–1.52)		1.08 (1.08–1.09)		0.95 (0.93–0.97)		0.75 (0.74–0.76)	
Denmark	1.70 (1.66–1.73)		1.36 (1.35–1.37)		1.28 (1.24–1.32)		1.49 (1.48–1.51)	
*Turkish-born*								
Netherlands	1.0 (ref.)	1.13 (1.06–1.22)	1.0 (ref.)	1.18 (1.14–1.23)	1.0 (ref.)	0.85 (0.77–0.95)	1.0 (ref.)	0.90 (0.84–0.96)
France	0.76 (0.64–0.89)	0.57 (0.49–0.67)	0.61 (0.56–0.67)	0.63 (0.58–0.69)	0.63 (0.48–0.83)	0.56 (0.43–0.72)	0.57 (0.49–0.67)	0.64 (0.55–0.74)
Denmark	1.28 (1.03–1.59)	0.89 (0.72–1.09)	2.22 (1.98–2.49)	1.88 (1.69–2.09)	1.54 (1.13–2.10)	1.07 (0.80–1.43)	2.41 (2.00–2.91)	1.47 (1.24–1.75)
*Moroccan-born*								
Netherlands	1.0 (ref.)	1.12 (1.03–1.21)	1.0 (ref.)	0.73 (0.70–0.77)	1.0 (ref.)	0.96 (0.86–1.08)	1.0 (ref.)	0.78 (0.71–0.84)
France	0.90 (0.80–1.02)	0.65 (0.59–0.71)	0.92 (0.86–0.97)	0.62 (0.60–0.64)	0.74 (0.62–0.88)	0.70 (0.61–0.80)	0.79 (0.72–0.87)	0.79 (0.75–0.84)
Denmark	1.93 (1.33–2.80)	1.34 (0.93–1.92)	2.28 (1.69–3.08)	1.29 (0.96–1.74)	1.18 (0.56–2.49)	0.93 (0.44–1.95)	1.58 (0.85–2.95)	0.84 (0.45–1.56)

^a^The reference group was the local-born population in the respective country of residence.

^b^Mortality rate ratios were adjusted for age. MRR = mortality rate ratios. CI = confidence interval.

Age- and sex-adjusted analyses by main cause of death are shown in Table 4. Mortality for suicide, respiratory diseases, cancer and unintentional injuries were generally lower in immigrants compared to local-born, with little cross-country differences. Homicide mortality was especially higher for both Turkish-born and Moroccan-born in the Netherlands as compared to local-born, and a similar pattern, with smaller differences, held for infectious diseases. Compared to their peers in the Netherlands, CVD mortality for the immigrants was lowest in France and highest in Denmark (significant for Turkish-born only). Compared to the local-born, Turkish-born in Denmark and the Netherlands had higher CVD mortality, while Moroccan-born had lower mortality in the Netherlands and France. Finally, mortality due to other causes was especially lower for both Turkish-born and Moroccan-born living in France and higher for those living in Denmark as compared to their peers in the Netherlands.

**Table 4:** Cause-specific mortality rate ratios (MRR) comparing with the local-born population^a^ and with peers in the Netherlands among Turkish- and Moroccan-born, both men and women.

	**Suicide**		**Homicide**		**Cardiovascular diseases** ^c^		**Respiratory diseases** ^d^	
	MRR^b^ vs. peers	MRR^b^ vs. local-born	MRR^b^ vs. peers	MRR^b^ vs. local-born	MRR^b^ vs. peers	MRR^b^ vs. local-born	MRR^b^ vs. peers	MRR^b^ vs. local-born
*Local-born*								
Netherlands	1.0 (ref.)		1.0 (ref.)		1.0 (ref.)		1.0 (ref.)	
France	1.84 (1.80–1.88)		0.82 (0.75–0.91)		0.58 (0.58–0.59)		0.82 (0.81–0.83)	
Denmark	1.64 (1.59–1.69)		1.50 (1.35–1.68)		1.33 (1.31–1.34)		1.37 (1.34–1.39)	
*Turkish-born*								
Netherlands	1.0 (ref.)	0.62 (0.52–0.73)	1.0 (ref.)	6.15 (5.11–7.40)	1.0 (ref.)	1.15 (1.08–1.21)	1.0 (ref.)	0.67 (0.61–0.74)
France	0.91 (0.64–1.30)	0.29 (0.21–0.40)	0.48 (0.21–1.08)	1.40 (0.66–2.94)	0.56 (0.49–0.64)	1.04 (0.92–1.18)	0.91 (0.75–1.11)	0.65 (0.54–0.77)
Denmark	0.91 (0.52–1.61)	0.42 (0.24–0.72)	0.18 (0.09–0.39)	1.92 (0.86–4.31)	1.93 (1.62–2.28)	1.77 (1.51–2.08)	1.41 (0.99–1.99)	0.77 (0.55–1.07)
*Moroccan-born*								
Netherlands	1.0 (ref.)	0.53 (0.43–0.64)	1.0 (ref.)	5.83 (4.74–7.18)	1.0 (ref.)	0.73 (0.68–0.79)	1.0 (ref.)	0.54 (0.48–0.61)
France	1.44 (1.12–1.85)	0.38 (0.33–0.44)	0.22 (0.13–0.35)	1.26 (0.81–1.94)	0.60 (0.55–0.66)	0.75 (0.70–0.79)	1.13 (0.99–1.29)	0.65 (0.60–0.69)
Denmark	1.32 (0.42–4.16)	0.48 (0.16–1.50)	1.22 (0.39–3.83)	5.00 (1.61–15.58)	1.25 (0.69–2.26)	0.74 (0.41–1.34)	1.49 (0.66–3.34)	0.80 (0.36–1.78)
	**Infectious diseases** ^e^		**Cancer** ^f^		**Unintentional injuries** ^**g**^		**Other causes**	
	MRR^b^ vs. peers	MRR^b^ vs. local-born	MRR^b^ vs. peers	MRR^b^ vs. local-born	MRR^b^ vs. peers	MRR^b^ vs. local-born	MRR^b^ vs. peers	MRR^b^ vs. local-born
*Local-born*								
Netherlands	1.0 (ref.)		1.0 (ref.)		1.0 (ref.)		1.0 (ref.)	
France	1.52 (1.46–1.58)		0.97 (0.97–0.98)		2.00 (1.96–2.05)		1.52 (1.50–1.54)	
Denmark	1.58 (1.50–1.67)		1.20 (1.18–1.21)		2.34 (2.28–2.40)		1.92 (1.90–1.95)	
*Turkish-born*								
Netherlands	1.0 (ref.)	1.48 (1.18–1.84)	1.0 (ref.)	0.63 (0.60–0.67)	1.0 (ref.)	0.81 (0.70–0.92)	1.0 (ref.)	2.36 (2.24–2.48)
France	0.84 (0.54–1.33)	0.83 (0.55–1.23)	1.01 (0.90–1.13)	0.62 (0.56–0.68)	1.38 (1.07–1.77)	0.58 (0.47–0.72)	0.31 (0.26–0.36)	0.45 (0.38–0.52)
Denmark	1.39 (0.64–3.01)	0.77 (0.37–1.61)	1.26 (1.01–1.56)	0.70 (0.57–0.86)	1.00 (0.62–1.60)	0.35 (0.23–0.56)	3.02 (2.67–3.43)	3.45 (3.08–3.87)
*Moroccan-born*								
Netherlands	1.0 (ref.)	1.72 (1.38–2.15)	1.0 (ref.)	0.52 (0.49–0.56)	1.0 (ref.)	0.97 (0.84–1.11)	1.0 (ref.)	1.69 (1.58–1.80)
France	0.83 (0.63–1.10)	0.98 (0.83–1.16)	1.39 (1.29–1.51)	0.68 (0.66–0.71)	1.55 (1.30–1.85)	0.71 (0.64–0.78)	0.56 (0.51–0.61)	0.60 (0.57–0.64)
Denmark	1.62 (0.40–6.59)	1.08 (0.27–4.30)	1.70 (1.08–2.68)	0.84 (0.54–1.32)	2.15 (1.14–4.06)	0.96 (0.52–1.78)	2.90 (2.12–3.97)	2.46 (1.81–3.34)

MRR = mortality rate ratios. CI = confidence interval.

^a^The reference group was the local-born population in the respective country of residence.

^b^Mortality rate ratios were adjusted for age and sex.

^c^Cardiovascular diseases include hypertension, ischaemic heart disease, chronic rheumatic heart disease, other heart disease, cerebrovascular disease, other circulatory disease and diabetes.

^d^Respiratory diseases include COPD, asthma, pneumonia, influenza, and lung cancer.

^e^Infectious diseases includes HIV and TB.

^f^Cancer denotes total cancer mortality including lung cancer.

^g^Unintentional injuries are traffic and non-traffic injuries and other external causes.

## Discussion

This study aimed to assess differences in mortality between immigrants residing in countries with different integration policy models. We found that in Turkish- and Moroccan-born immigrants all-cause mortality was highest in Denmark (exclusionist model), followed by the Netherlands (multiculturalist) and France (assimilationist). Further, compared with the local-born population, immigrants in Denmark had higher mortality pattern while those in France had lower mortality. These patterns were generally more pronounced in the Turkish-born and older age group, but similar across sexes. By main cause of death, these patterns were, to some extent, observed for mortality of cardiovascular diseases (CVD), but not for suicide and homicide.

### Limitations

This study had several limitations. First, methods of data collection differed across the countries. For the Netherlands and Denmark we used linked data collected for around ten years while for France unlinked data from two years. Previous studies have raised concerns about the underestimation of mortality of immigrants [24,25] due to phenomena labelled as “mobility bias”, where frequent home country returns or even remigrations go unregistered thus inflating the time or population at risk [26,27]. Some of these problems are more likely to affect unlinked data due to a discrepancy between the mortality register (numerator) and population census (denominator) [28]. However, unlinked data may also suffer from an overestimation as deaths of irregular immigrants, who are absent in population registers, are otherwise recorded in the national mortality statistics [24].

The inclusion only of immigrants enumerated in the population registers limits generalisation of findings to undocumented migrants. It should be noted, however, that this group is arguably less affected by integration policies covered by MIPEX and more by immigration control policies [29].

The sample sizes for the immigrant populations in Denmark were rather small, particularly for the Moroccan-born. The pattern of higher mortality for immigrants in Denmark may therefore be more accurately assessed in the Turkish-born than the Moroccan-born. In a recent publication by Statistics Denmark, Turkish-born mortality in 2005–2009 was slightly lower than for native Danes [30]. However, the discrepancy with our results may be explained by the fact that this other study used unlinked data, included population aged until 89 years and excluded deaths outside Denmark.

An innovation of this study is the possibility to compare immigrants born in the same country that live in different European countries. Still, despite the common origin, there might be unmeasured confounding regarding both pre- and post-migration factors. As noted in the Introduction, as far as we know, the socioeconomic background, reasons for migration and regions of origin were fairly similar for Turkish and Moroccan immigrants to the three countries [18,19]. As such, we don’t expect important differences in pre-migration risk factors, including poverty, diet or other health-related behaviours. We lack measures of socioeconomic conditions or cardiovascular risk factors in the host country, which could partially explain inequalities between natives and immigrants [9]. However, these conditions should not be regarded as confounders but as potential intermediary factors between integration policies and health. An earlier study showed that these conditions were poorer for immigrants in “exclusionist” countries [16].

### Potential relationship with policies

Residence in Denmark, a country with a pattern of integration policy that can be classified as “differential exclusionist” [5], is associated with relatively unfavourable mortality pattern among immigrants compared to both local-born Danes and immigrants elsewhere. This corresponds to the findings of previous cross-country comparisons of self-reported health among non-EU immigrants [16] and neonatal mortality for offspring of Turkish mothers [31]. Denmark became a net immigration country in the 1950s, when it started receiving labour migration, mainly from Turkey and Yugoslavia, but at a small scale compared to its neighbours. In the 1980s, when legal reforms increased the possibilities of family reunification and asylum, Denmark immigration policy became pronouncedly humanitarian. However, since 1992 this legislation was progressively restricted, increasing the requirements for permanent residence and reunification, including tests on Danish language and the signature of an integration contract [32]. Nowadays, Denmark generally performs worse than France and especially the Netherlands across multiple indicators of integration policy [6] and outcomes such as social tolerance [2], immigrants' experience of discrimination [33], naturalisation rates and material standards of living [16,34] (see S2 Table for a selection of these indicators).

In contrast with our hypothesis, and with the rather poor socioeconomic outcomes for immigrants in France (S2 Table), we found that residence in France, the “assimilationist” country, was associated with the lowest all-cause mortality in the Turkish- and Moroccan-born. In a previous cross-country comparison of CVD mortality with MEHO data, the low mortality for both local-born and immigrants in France as compared to other countries was viewed as an extension of the "French paradox" [23]. However in the present study, immigrants to France were also found to have the lowest mortality risk as compared to local-born in France. A recent study on self-rated health found that immigrants had poorer health than natives both in France and in the Netherlands, and that ethnic inequalities were greatest among women in the Netherlands [16]. As commented in the Introduction, the adhesion of countries to policy models has not been unequivocal and rigid over time [3]. While France has been slightly moving from assimilationism to inclusiveness and multiculturalism, the Netherlands has walked the opposite path. The Dutch 1998 integration of newcomers act was considered a sharp critique to multiculturalism, while the 2003 citizenship act introduced integration tests for naturalisation [35]. Moreover, Turkish and Moroccan migrants in the Netherlands have historically received a less inclusive, “guest workers’” treatment as compared to migrants from former Dutch colonies [36].

We hypothesised that the correspondence between integration policy model and immigrants’ mortality would be greater for causes of death that are more sensitive to material and psychosocial conditions and larger socioeconomic inequalities in mortality. We found indeed higher mortality due to cardiovascular and respiratory diseases for immigrants in Denmark. However, this partly reflected the pattern in the local-born population, suggesting a shared exposure to adverse environmental factors [37]. As this is an explorative study, we grouped causes of death rather broadly: future studies may dig into more specific causes to understand specific pathways.

### Alternative explanations

We should acknowledge that differential exposures in the host country other than the policy environment might also explain the mortality patterns observed in this study. First, previous studies have related ethnic density with reduced mortality in US Blacks and Hispanics [38]. It may be relevant that the share of Turkish and Moroccans on the total population is lower in Denmark than in the Netherlands. However, it is also low for Turkish in France, who show an even more favourable mortality pattern than the much “denser” Moroccans.

Second, it is often argued that mortality rates of immigrant populations are strongly determined by health selection processes both at immigration (the “healthy migrant effect”) and at remigration (the “salmon bias” or “unhealthy remigrant effect”), although a recent series of Danish studies has cast doubts on their real extent [39,40]. Cross-national variations in the mortality rates of Turkish or Moroccan immigrants could be influenced by variations in the strength of such selection effects. Though such variations are possible in theory (e.g. because of varying distance to the country of origin), we have no data to support this possibility.

Third, the uptake of the western lifestyle and related cardiovascular risk factors such as diet or smoking are important in shaping immigrants’ mortality risk. As most immigrants have lived for many decades in their destination countries, it is not surprising that their mortality differences across countries of residence partly mirror those of the local-born population [23]. However, we have also found country differences in inequalities between immigrants and local populations. Previous reviews have shown that in the Netherlands, both Turkish and Moroccan men and women had in general higher metabolic risk factors [41], and Turkish men had high prevalence of smoking [42], which may explain our finding of higher mortality in these two groups as compared to the native Dutch. Similar studies from France and Denmark are lacking, besides one study showing high prevalence of diabetes among Turkish in Denmark [40] and a healthier diet and similar smoking levels as natives among Moroccans in France [43]. Nevertheless, it is important to consider that these behaviours can be a response to unfavourable material and psychosocial conditions [7,44].

Similarly, differences in healthcare access are another factor resulting from different integration policies that might explain the cross-country differences in immigrants’ mortality. However, in the three countries we studied, legally registered immigrants have the same rights in access to healthcare as country nationals [45]. Cross-country studies on healthcare access and use of immigrant populations are lacking, and in a systematic review of such studies at the country level, no French study was identified, while Danish and Dutch studies found similar patterns of higher GP use for immigrants [46]. As such, we cannot conclude that healthcare access is likely to be an important explanatory factor.

### Conclusions and further research

We found that residence in Denmark, a country with an “exclusionist” integration model, is associated with the highest mortality rates for immigrants from Turkey and Morocco, followed by the “inclusive” Netherlands and then the “assimilationist” France. This pattern was particularly observed among Turkish-born immigrants, in the age group 45–69 years, and for mortality due to cardiovascular diseases. Problems of data comparability and unmeasured confounding restrict our ability to make causal inferences on the role of different policies. Yet, these findings, combined with previous comparative studies [16,31] may be a wake-up call for Danish authorities to consider the possibility that the restrictive turn in the immigration-related policy, politics and social climate [32] might have contributed to higher mortality rates of Moroccan and Turkish immigrants.

This study is explorative and encourages more research in several ways. First, this study underlines the need for comparable mortality registration systems across Europe, including detailed socio-demographic information and reason for migration of immigrant populations. Second, it shows the potential of conducting cross-national comparisons on immigrant populations with same origins to raise hypotheses on the health impact of different host country environments. Third, such studies could benefit from the inclusion of classical immigration countries such as US, Australia, Canada as they represent yet another integration model—namely the “pluralist”, which is absent in Europe [47,48]. Fourth, while in this study we assessed broad integration models, further studies should assess the associations of specific aspects of integration policies, including healthcare policies, with immigrants' health.

## Supporting Information

S1 Table Cause-specific mortality rate ratios (MRR) comparing with the local-born population^a^ and with peers in the Netherlands among Turkish- and Moroccan-born immigrants. MRR = mortality rate ratios. CI = confidence interval.
(DOCX)

S2 Table Overview of policy indicators and outcomes in France, the Netherlands, and Denmark. MIPEX = Migrant Integration Policy Index [6]. OECD = Organisation for Economic Cooperation and Development [34]. EU-MIDIS = European Union Minorities and Discrimination Survey [33]. Numbers in brackets indicate the difference compared to natives, or to the total population (brackets and italics).
(DOCX)
