# Supplementary material for: Association between Integration Policies and Immigrants’ Mortality: An Explorative Study across Three European Countries
Source: PLoS One. 2015 Jun 12;10(6):e0129916. doi: 10.1371/journal.pone.0129916 (PMC4466572; doi:10.1371/journal.pone.0129916)
Supplement: S1 Table — MRR = mortality rate ratios. CI = confidence interval. (DOCX) [file pone.0129916.s001.docx]

**S1A Table. Cause-specific mortality rate ratios (MRR) comparing with the local-born population^a^ and with peers in the Netherlands among Turkish- and Moroccan-born men.**

|  | **Suicide** | | **Homicide** | | **Cardiovascular diseases**^c^ | | **Respiratory diseases**^d^ | |
| --- | --- | --- | --- | --- | --- | --- | --- | --- |
|  | MRR^b^ vs. peers | MRR^b^ vs. local-born | MRR^b^ vs. peers | MRR^b^ vs. local-born | MRR^b^ vs. peers | MRR^b^ vs. local-born | MRR^b^ vs. peers | MRR^b^ vs. local-born |
| *Local-born* |  |  |  |  |  |  |  |  |
| Netherlands | 1.0 (ref.) |  | 1.0 (ref.) |  | 1.0 (ref.) |  | 1.0 (ref.) |  |
| France | 2.02 (1.97-2.08) |  | 0.80 (0.71-0.90) |  | 0.63 (0.62-0.64) |  | 1.02 (1.00-1.03) |  |
| Denmark | 1.71 (1.65-1.78) |  | 1.35 (1.17-1.55) |  | 1.32 (1.30-1.33) |  | 1.14 (1.12-1.16) |  |
| *Turkish-born* |  |  |  |  |  |  |  |  |
| Netherlands | 1.0 (ref.) | 0.68 (0.57-0.82) | 1.0 (ref.) | 7.38 (5.99-9.09) | 1.0 (ref.) | 1.15 (1.07-1.23) | 1.0 (ref.) | 0.83 (0.75-0.92) |
| France | 0.83 (0.55-1.26) | 0.26 (0.18-0.38) | 0.22 (0.10-0.48) | 1.96 (0.93-4.14) | 0.56 (0.48-0.65) | 0.98 (0.85-1.13) | 0.92 (0.75-1.14) | 0.70 (0.58-0.83) |
| Denmark | 0.78 (0.39-1.54) | 0.37 (0.19-0.71) | 0.19 (0.05-0.78) | 0.99 (0.25-4.00) | 1.80 (1.47-2.20) | 1.66 (1.37-2.01) | 1.25 (0.84-1.85) | 0.98 (0.67-1.43) |
| *Moroccan-born* |  |  |  |  |  |  |  |  |
| Netherlands | 1.0 (ref.) | 0.60 (0.48-0.74) | 1.0 (ref.) | 6.38 (5.01-8.11) | 1.0 (ref.) | 0.67 (0.62-0.74) | 1.0 (ref.) | 0.63 (0.56-0.71) |
| France | 1.29 (0.97-1.71) | 0.36 (0.30-0.43) | 0.22 (0.13-0.38) | 1.46 (0.89-2.41) | 0.66 (0.59-0.74) | 0.70 (0.66-0.75) | 1.11 (0.97-1.28) | 0.66 (0.61-0.71) |
| Denmark | 1.00 (0.25-4.08) | 0.39 (0.10-1.56) | 1.03 (0.25-4.21) | 4.87 (1.21-19.58) | 1.02 (0.49-2.16) | 0.59 (0.28-1.25) | 1.67 (0.74-3.75) | 1.24 (0.56-2.77) |
| Continued… | | | | | | | | |
|  | **Infectious diseases**^e^ | | **Cancer**^f^ | | **Unintentional injuries** | | **Other causes** | |
|  | MRR^b^ vs. peers | MRR^b^ vs. local-born | MRR^b^ vs. peers | MRR^b^ vs. local-born | MRR^b^ vs. peers | MRR^b^ vs. local-born | MRR^b^ vs. peers | MRR^b^ vs. local-born |
| *Local-born* |  |  |  |  |  |  |  |  |
| Netherlands | 1.0 (ref.) |  | 1.0 (ref.) |  | 1.0 (ref.) |  | 1.0 (ref.) |  |
| France | 1.68 (1.59-1.76) |  | 1.17 (1.16-1.18) |  | 2.05 (2.00-2.10) |  | 1.75 (1.73-1.78) |  |
| Denmark | 1.87 (1.75-1.99) |  | 1.10 (1.09-1.12) |  | 2.25 (2.18-2.33) |  | 2.02 (1.99-2.06) |  |
| *Turkish-born* |  |  |  |  |  |  |  |  |
| Netherlands | 1.0 (ref.) | 1.53 (1.19-1.98) | 1.0 (ref.) | 0.73 (0.68-0.79) | 1.0 (ref.) | 0.64 (0.45-0.90) | 1.0 (ref.) | 2.51 (2.36-2.67) |
| France | 0.64 (0.35-1.14) | 0.62 (0.37-1.05) | 1.04 (0.91-1.19) | 0.63 (0.56-0.71) | 1.48 (1.13-1.94) | 0.64 (0.51-0.80) | 0.32 (0.27-0.39) | 0.44 (0.37-0.52) |
| Denmark | 1.57 (0.68-3.66) | 0.79 (0.35-1.76) | 1.23 (0.94-1.60) | 0.84 (0.66-1.09) | 0.91 (0.53-1.54) | 0.35 (0.21-0.57) | 3.15 (2.72-3.65) | 3.54 (3.10-4.04) |
| *Moroccan-born* |  |  |  |  |  |  |  |  |
| Netherlands | 1.0 (ref.) | 1.43 (1.08-1.89) | 1.0 (ref.) | 0.54 (0.50-0.59) | 1.0 (ref.) | 0.82 (0.58-1.16) | 1.0 (ref.) | 1.62 (1.49-1.75) |
| France | 1.03 (0.73-1.46) | 0.90 (0.74-1.11) | 1.40 (1.27-1.54) | 0.62 (0.60-0.65) | 1.55 (1.28-1.88) | 0.72 (0.65-0.81) | 0.61 (0.55-0.68) | 0.56 (0.52-0.60) |
| Denmark | 2.33 (0.57-9.61) | 1.22 (0.31-4.89) | 2.39 (1.49-3.83) | 1.32 (0.83-2.10) | 2.44 (1.29-4.61) | 1.14 (0.61-2.12) | 3.14 (2.19-4.52) | 2.37 (1.67-3.37) |

MRR=mortality rate ratios. CI=confidence interval.

^a^The reference group was the local-born population in the respective country of residence.

^b^Mortality rate ratios were adjusted for age.

^c^Cardiovascular diseases also include diabetes mortality.

^d^Respiratory diseases include COPD, asthma, pneumonia, influenza, and lung cancer.

^e^Infectious diseases includes HIV and TB.

^f^Cancer denotes total cancer mortality including lung cancer.

**S1B Table. Cause-specific mortality rate ratios (MRR) comparing with the local-born population^a^ and with peers in the Netherlands among Turkish- and Moroccan-born women.**

|  | **Suicide** | | **Homicide** | | **Cardiovascular diseases**^c^ | | | **Respiratory diseases**^d^ | | |
| --- | --- | --- | --- | --- | --- | --- | --- | --- | --- | --- |
|  | MRR^b^ vs. peers | MRR^b^ vs. local-born | MRR^b^ vs. peers | MRR^b^ vs. local-born | MRR^b^ vs. peers | MRR^b^ vs. local-born | | MRR^b^ vs. peers | MRR^b^ vs. local-born | |
| *Local-born* |  |  |  |  |  |  | |  |  | |
| Netherlands | 1.0 (ref.) |  | 1.0 (ref.) |  | 1.0 (ref.) |  | | 1.0 (ref.) |  | |
| France | 1.46 (1.40-1.52) |  | 0.87 (0.74-1.03) |  | 0.48 (0.47-0.49) |  | | 0.49 (0.47-0.50) |  | |
| Denmark | 1.48 (1.40-1.56) |  | 1.85 (1.54-2.22) |  | 1.35 (1.32-1.37) |  | | 1.78 (1.74-1.82) |  | |
| *Turkish-born* |  |  |  |  |  |  | |  |  | |
| Netherlands | 1.0 (ref.) | 0.46 (0.33-0.66) | 1.0 (ref.) | 3.50 (2.32-5.29) | 1.0 (ref.) | 1.14 (1.02-1.27) | | 1.0 (ref.) | 0.28 (0.22-0.37) | |
| France | 1.24 (0.62-2.48) | 0.38 (0.21-0.70) | – | – | 0.56 (0.42-0.73) | 1.27 (0.99-1.64) | | 0.80 (0.44-1.44) | 0.39 (0.23-0.66) | |
| Denmark | 1.42 (0.50-4.04) | 0.60 (0.22-1.59) | 1.77 (0.61-5.11) | 3.62 (1.34-9.78) | 2.33 (1.70-3.19) | 2.09 (1.55-2.81) | | 2.43 (1.15-5.13) | 0.45 (0.23-0.91) | |
| *Moroccan-born* |  |  |  |  |  |  | |  |  | |
| Netherlands | 1.0 (ref.) | 0.33 (0.21-0.53) | 1.0 (ref.) | 4.48 (2.96-6.78) | 1.0 (ref.) | 0.93 (0.81-1.07) | | 1.0 (ref.) | 0.26 (0.19-0.36) | |
| France | 2.07 (1.18-3.65) | 0.45 (0.35-0.59) | 0.20 (0.08-0.55) | 0.90 (0.37-2.17) | 0.46 (0.38-0.55) | 0.91 (0.81-1.03) | | 1.23 (0.84-1.80) | 0.56 (0.47-0.67) | |
| Denmark | 3.27 (0.44-24.63) | 0.93 (0.13-6.64) | 1.87 (0.25-13.82) | 5.27 (0.74-37.69) | 1.77 (0.66-4.76) | 1.27 (0.48-3.40) | | – | – | |
| Continued… | | | | | | | | | | |
|  | **Infectious diseases**^e^ | | **Cancer**^f^ | | **Unintentional injuries** | | | **Other causes** | | |
|  | MRR^b^ vs. peers | MRR^b^ vs. local-born | MRR^b^ vs. peers | MRR^b^ vs. local-born | MRR^b^ vs. peers | | MRR^b^ vs. local-born | MRR^b^ vs. peers | | MRR^b^ vs. local-born |
| *Local-born* |  |  |  |  |  | |  |  | |  |
| Netherlands | 1.0 (ref.) |  | 1.0 (ref.) |  | 1.0 (ref.) | |  | 1.0 (ref.) | |  |
| France | 1.22 (1.14-1.31) |  | 0.74 (0.73-0.75) |  | 1.87 (1.79-1.95) | |  | 1.17 (1.14-1.19) | |  |
| Denmark | 1.04 (0.94-1.15) |  | 1.30 (1.29-1.32) |  | 2.59 (2.45-2.72) | |  | 1.77 (1.72-1.81) | |  |
| *Turkish-born* |  |  |  |  |  | |  |  | |  |
| Netherlands | 1.0 (ref.) | 1.34 (0.87-2.07) | 1.0 (ref.) | 0.48 (0.43-0.53) | 1.0 (ref.) | | 0.64 (0.45-0.90) | 1.0 (ref.) | | 2.06 (1.88-2.26) |
| France | 1.52 (0.72-3.24) | 1.55 (0.83-2.88) | 0.94 (0.76-1.17) | 0.58 (0.48-0.70) | 0.82 (0.38-1.79) | | 0.30 (0.15-0.60) | 0.28 (0.21-0.39) | | 0.47 (0.35-0.64) |
| Denmark | 0.77 (0.10-5.76) | 0.64 (0.90-4.55) | 1.32 (0.91-1.92) | 0.53 (0.37-0.76) | 1.57 (0.55-4.46) | | 0.39 (0.15-1.04) | 2.72 (2.14-3.45) | | 3.21 (2.58-4.00) |
| *Moroccan-born* |  |  |  |  |  | |  |  | |  |
| Netherlands | 1.0 (ref.) | 2.62 (1.83-3.75) | 1.0 (ref.) | 0.47 (0.42-0.53) | 1.0 (ref.) | | 0.82 (0.58-1.16) | 1.0 (ref.) | | 1.85 (1.65-2.07) |
| France | 0.54 (0.33-0.86) | 1.25 (0.93-1.69) | 1.37 (1.19-1.58) | 0.81 (0.76-0.86) | 1.51 (0.98-2.31) | | 0.70 (0.56-0.86) | 0.46 (0.39-0.54) | | 0.72 (0.65-0.80) |
| Denmark | – | – | 0.29 (0.04-2.09) | 0.12 (0.02-0.86) | – | | – | 2.30 (1.22-4.32) | | 2.62 (1.41-4.87) |

MRR=mortality rate ratios. CI=confidence interval.

^a^The reference group was the local-born population in the respective country of residence.

^b^Mortality rate ratios were adjusted for age.

^c^Cardiovascular diseases also include diabetes mortality.

^d^Respiratory diseases include COPD, asthma, pneumonia, influenza, and lung cancer.

^e^Infectious diseases includes HIV, TB and other infections.

^f^Cancer denotes total cancer mortality including lung cancer.
