# Supplementary material for: Association between Integration Policies and Immigrants’ Mortality: An Explorative Study across Three European Countries
Source: PLoS One. 2015 Jun 12;10(6):e0129916. doi: 10.1371/journal.pone.0129916 (PMC4466572; doi:10.1371/journal.pone.0129916)
Supplement: S2 Table — MIPEX = Migrant Integration Policy Index [6]. OECD = Organisation for Economic Cooperation and Development [34]. EU-MIDIS = European Union Minorities and Discrimination Survey [33]. Numbers in brackets indicate the difference compared to natives, or to the total population (brackets and italics). (DOCX) [file pone.0129916.s002.docx]

**S2 Table. Overview of policy indicators and outcomes in France, the Netherlands, and Denmark.**

| **Dimension** | **Indicator** | **Source** | **Year of data** | **Denmark** | **France** | **Netherlands** |
| --- | --- | --- | --- | --- | --- | --- |
| *Policy indicators* | | | | | | |
| Integration policy | MIPEX total score (0 worst, 100 best) | MIPEX | 2007 | 51 | 54 | 71 |
| Legal status | Long-term residence policy score | MIPEX | 2007 | 64 | 46 | 68 |
| Naturalisation | Access to nationality score | MIPEX | 2007 | 33 | 59 | 65 |
| Discrimination | Anti-discrimination policy score | MIPEX | 2007 | 42 | 74 | 68 |
| *Policy outcomes* | | | | | | |
| Naturalisation | % naturalised foreign-born (non-recent) | [OECD](http://dx.doi.org/10.1787/888932736509) | 2005-06 | 52 | 60 | 76 |
| Population attitudes | Social tolerance (0 intolerant, 100 tolerant) | Eurobarometer [2] | 1997 | 51 | 55 | 72 |
| Discrimination | % Discriminated against last year | [EU-MIDIS](http://fra.europa.eu/sites/default/files/fra_uploads/663-FRA-2011_EU_MIDIS_EN.pdf) | 2008 | 42 | 25 | 30 |
| Socioeconomic segregation | % Low-skilled level of employment | [OECD](http://dx.doi.org/10.1787/888932736566) | 2009-11 | 15 *(7)* | 18 *(9)* | 15 *(10)* |
| Employment conditions | % Unemployment rate (born in low-income country, aged 15-64) | [OECD](http://dx.doi.org/10.1787/888932735255) | 2009-10 | 13 (7) | 17 (9) | 9 (5) |
| Material standards | % At risk of poverty or social exclusion (non-EU born aged 25-54) | [Eurostat](http://epp.eurostat.ec.europa.eu/portal/page/portal/employment_social_policy_equality/documents/Soc_incl_2009_2010_2011_COB.xls) [47] | 2008 | 36 (23) | 40 (26) | 25 (12) |
| Material standards | % Households in lowest decile of income | [OECD](http://dx.doi.org/10.1787/888932736623) | 2008 | 32 | 27.8 | 30.4 |
| Material standards | % living in a deprived dwelling | [OECD](http://dx.doi.org/10.1787/888932736167) | 2009 | 10 (0) | 9 (4) | 8 (5) |

MIPEX=Migrant Integration Policy Index [6]. OECD=Organisation for Economic Cooperation and Development [36]. EU-MIDIS=European Union Minorities and Discrimination Survey [35].

Numbers in brackets indicate the difference compared to natives, or to the total population (brackets and italics).
